# Group A Streptococcus pharyngitis and pharyngeal carriage: A meta-analysis

**DOI:** 10.1371/journal.pntd.0006335

**Published:** 2018-03-19

**Authors:** Jane Oliver, Erandi Malliya Wadu, Nevil Pierse, Nicole J. Moreland, Deborah A. Williamson, Michael G. Baker

**Affiliations:** 1 University of Otago Wellington, Newtown, Wellington, New Zealand; 2 Maurice Wilkins Centre and School of Medical Sciences, University of Auckland, Auckland, New Zealand; 3 Department of Microbiology and Immunology, University of Melbourne, Peter Doherty Institute for Infection and Immunity, Melbourne, Australia; King Saud University College of Medicine, SAUDI ARABIA

## Abstract

**Objective:**

Antibiotic treatment of Group A Streptococcus (GAS) pharyngitis is important in acute rheumatic fever (ARF) prevention, however clinical guidelines for prescription vary. GAS carriers with acute viral infections may receive antibiotics unnecessarily. This review assessed the prevalence of GAS pharyngitis and carriage in different settings.

**Methods:**

A random-effects meta-analysis was performed. Prevalence estimates for GAS+ve pharyngitis, serologically-confirmed GAS pharyngitis and asymptomatic pharyngeal carriage were generated. Findings were stratified by age group, recruitment method and country income level. Medline and EMBASE databases were searched for relevant literature published between 1 January 1946 and 7 April 2017. Studies reporting prevalence data on GAS+ve or serologically-confirmed GAS pharyngitis that stated participants exhibited symptoms of pharyngitis or upper respiratory tract infection (URTI) were included. Included studies reporting the prevalence of asymptomatic GAS carriage needed to state participants were asymptomatic.

**Results:**

285 eligible studies were identified. The prevalence of GAS+ve pharyngitis was 24.1% (95% CI: 22.6–25.6%) in clinical settings (which used ‘passive recruitment’ methods), but less in sore throat management programmes (which used ‘active recruitment’, 10.0%, 8.1–12.4%). GAS+ve pharyngitis was more prevalent in high-income countries (24.3%, 22.6–26.1%) compared with low/middle-income countries (17.6%, 14.9–20.7%). In clinical settings, approximately 10% of children swabbed with a sore throat have serologically-confirmed GAS pharyngitis, but this increases to around 50–60% when the child is GAS culture-positive. The prevalence of serologically-confirmed GAS pharyngitis was 10.3% (6.6–15.7%) in children from high-income countries and their asymptomatic GAS carriage prevalence was 10.5% (8.4–12.9%). A lower carriage prevalence was detected in children from low/middle income countries (5.9%, 4.3–8.1%).

**Conclusions:**

In active sore throat management programmes, if the prevalence of GAS detection approaches the asymptomatic carriage rate (around 6–11%), there may be little benefit from antibiotic treatment as the majority of culture-positive patients are likely carriers.

## Author summary

Treating sore throats caused by Group A Streptococcus infections (GAS pharyngitis) with antibiotics is important for preventing acute rheumatic fever (ARF). It is impossible to distinguish patients with true GAS pharyngitis infections from GAS carriers with pharyngitis caused by viral infections when throat swab culturing alone is used to diagnose GAS pharyngitis. Carriers are not likely to benefit from antibiotic treatment, but may receive treatment unnecessarily. Reported rates of GAS pharyngitis and carriage vary considerably depending on the setting. Thus it is difficult to ascertain which groups are likely to benefit significantly from active sore throat management programmes which treat GAS pharyngitis in order to prevent ARF. We performed a meta-analysis to estimate the prevalence of GAS pharyngitis and asymptomatic carriage in different settings. Approximately 10% of all children swabbed for a sore throat in clinical settings have true GAS pharyngitis, but this increases to around 55% if the children have GAS detected in their throat using swab cultures. In active sore throat management programmes, the prevalence of GAS detection is lower than in clinical settings and if it declines towards 8% (the asymptomatic carriage level), there may be little benefit in treating GAS culture-positive patients with antibiotics.

## Introduction

Acute pharyngitis is a common cause of doctor’s visits across the world.[1] Most pharyngitis episodes (40–80%) are caused by self-limiting viral infections. Group A Streptococcus (GAS) infection is the most common bacterial cause of pharyngitis, responsible for approximately15-30% of cases.[2] In a small minority of patients (0.3–3%), untreated GAS pharyngitis may trigger acute rheumatic fever (ARF).[3–5] ARF and its sequela, rheumatic heart disease (RHD), remain important public health problems in low- and middle-income countries,[6–8] and persist in certain (predominantly Indigenous-minority) groups in high-income countries.[9, 10] Indigenous Australians and New Zealand Māori and Pacific populations have among the highest rates of ARF in the world.[1]

There is conflicting pressure on clinicians to either prescribe antibiotics to patients with pharyngitis to reduce the risk of ARF, or to withhold prescriptions and minimise antibiotic-related harms.[11–14] Most high-income countries have national clinical guidelines on antibiotic treatment of GAS pharyngitis, but guidelines differ markedly in their recommendations.[15] For example, in North America, Finland and France, throat swabbing and prescribing antibiotics to patients with GAS culture-positive (GAS+ve) pharyngitis is recommended.[15–17] Conversely, antibiotic treatment is discouraged in other high-income countries, notably the United Kingdom, Belgium and the Netherlands.[15, 18] In New Zealand and Australia, clinical guidelines restrict swabbing and treatment to patients at high-risk of ARF.[16, 17] Specifically, in New Zealand it is recommended that antibiotic treatment begin immediately after a symptomatic high-risk patient presents to a healthcare provider, but be discontinued if GAS is not cultured. In this instance, patients may be exposed to several days of unnecessary treatment while laboratory results are generated.[19, 20] Despite clinical guidelines, healthcare practitioners sometimes prescribe antibiotics to relieve symptom duration, regardless of the patient’s ARF risk.[21]

Accurate diagnosis of true GAS pharyngitis infection remains a major barrier to effective ARF prevention. Some individuals carry GAS in the throat, but have no symptoms of infection nor an antibody response.[11, 22] The Infectious Diseases Society of America makes a strong recommendation against routine antibiotic treatment of carriers. This recommendation is based on evidence in the literature indicating that carriers are unlikely to transmit GAS pharyngitis, and face little or no risk of developing complications (including ARF).[23] In addition, a previous review estimated the prevalence of asymptomatic pharyngeal GAS carriage at 12% amongst school-aged children.[24] When throat culture alone is used to diagnose GAS pharyngitis, many patients prescribed antibiotics are likely suffering viral pharyngitis with coincidental GAS carriage.[25–27] The reference standard for determining whether true GAS pharyngitis is present requires both throat culture and serological testing to identify GAS+ve patients with elevated antibodies targeting conserved GAS antigens, streptolysin-O and deoxyribonuclease-B.[28, 29] However, serological testing relies on obtaining patient blood samples and thus is not routinely performed in primary care. This situation has resulted in a major knowledge gap with respect to the prevalence of true GAS pharyngitis.

There is an important need to ensure that clinicians target high-risk individuals with effective, evidence-based treatment strategies, particularly in an era of increasing antimicrobial resistance. Accurate prevalence estimates of serologically-confirmed GAS pharyngitis across different geographic and population settings are therefore needed to inform clinical practice and policy. Accordingly, this study aimed to use a systematic literature review to determine: (i) the prevalence of GAS culture-positive pharyngitis in different settings and populations; (ii) the prevalence of serologically-confirmed GAS pharyngitis in symptomatic GAS+ve individuals; and (iii) the prevalence of asymptomatic pharyngeal GAS carriage.

## Methods

### Ethics approval

No patient recruitment or other involvement in this study was required and consequently ethics approval was not needed. All data analysed were anonymised.

### Search strategy and selection criteria

A systematic literature review was conducted and reported in accordance with PRISMA guidelines.[30] No pre-existing review protocols for were identified, but the methodology was loosely based around that of a previously published meta-analysis by Shaikh *et al*.[31] A total of 17 literature searches on Medline and EMBASE databases were performed to identify articles containing prevalence data on GAS+ve pharyngitis, serologically-confirmed GAS pharyngitis and asymptomatic pharyngeal GAS carriage published between 1 January 1946 and 7 April 2017. Search terms on Medline included MeSHs: ‘Streptococcal Infections’ AND ‘Pharyngitis’. Search terms on Embase included MeSHs: ‘Streptococcus Group A’ AND ‘Pharyngitis’, also ‘Streptococcal pharyngitis’ AND ‘Streptococcus Group A’. Keyword searches using the terms: ‘GAS pharyngitis’ OR ‘streptococcal pharyngitis’ AND ‘ASO’ OR ‘anti-streptolysin’ OR ‘anti-DNase-B’ were employed on both databases. Keyword searches using the terms: ‘ASOT’ OR ‘ADBT’ OR ‘ADB’ AND ‘streptococc*’ were also performed. Search findings were limited to ‘Humans’. Further details of the search strategy are listed in the Methodology Appendix. Publications were catalogued using Endnote X7. A researcher (JO) screened the search results and applied the eligibility criteria. Eligible literature, identified in title or abstract screening, was obtained for full screening. Where systematic reviews were identified, prevalence studies they referenced were searched by title on Google Scholar or Medline, if published, and searched by title using the Google search engine if not published. Non-English language papers were screened using Google translator.

Explicit *a priori* inclusion and exclusion criteria were applied to assess article quality and reduce bias. Only studies using throat swab culture with an agar plate and incubator to detect GAS were included. Because we were interested in the point prevalence of GAS, longitudinal studies in which participants were swabbed multiple times had data from the first swab included only. When investigating the prevalence of GAS+ve pharyngitis, all studies that stated participants exhibited symptoms of pharyngitis or upper respiratory tract infection (URTI) were included where they presented to health practitioners who decided to obtain a throat swab. GAS pharyngitis demonstrates a wide range of clinical presentations[23, 32] and we aimed to maximise the number of relevant studies included. For studies investigating serologically-confirmed GAS pharyngitis, the same criteria applied and prevalence data were abstracted where the authors considered their findings provided serological confirmation. Information on the method of confirmation was noted where available. For included studies investigating the prevalence of asymptomatic GAS carriage, the authors needed to state participants did not have symptoms of pharyngitis or URTI when the throat swab was obtained, otherwise were excluded. Only studies that reported the number of participants and the number (or proportion) that produced GAS in throat cultures were included (and where applicable, the number of participants that were serologically-confirmed as having GAS pharyngitis). The country or countries recruitment was conducted in was also required to be discernible for inclusion, as was the participant recruitment method (active/passive). Studies were excluded if they were not likely to be representative of the general population, notably those conducted in outbreaks and other distinct settings (for example, from closed communities such as detention centres). We excluded studies that could not be translated to English.

### Data abstraction

Demographic and prevalence data were abstracted and entered on a spreadsheet (JO). This included the study citation, participant age group/s, country study sample was drawn from, number of cases, sample size, and recruitment period (where available). A second reviewer (EMW) independently abstracted prevalence data. Where abstracted data differed between the two reviewers, the article was rechecked and remaining differences resolved through discussion between the study authors.

### Grouping of results and definitions

**Fig 1:**
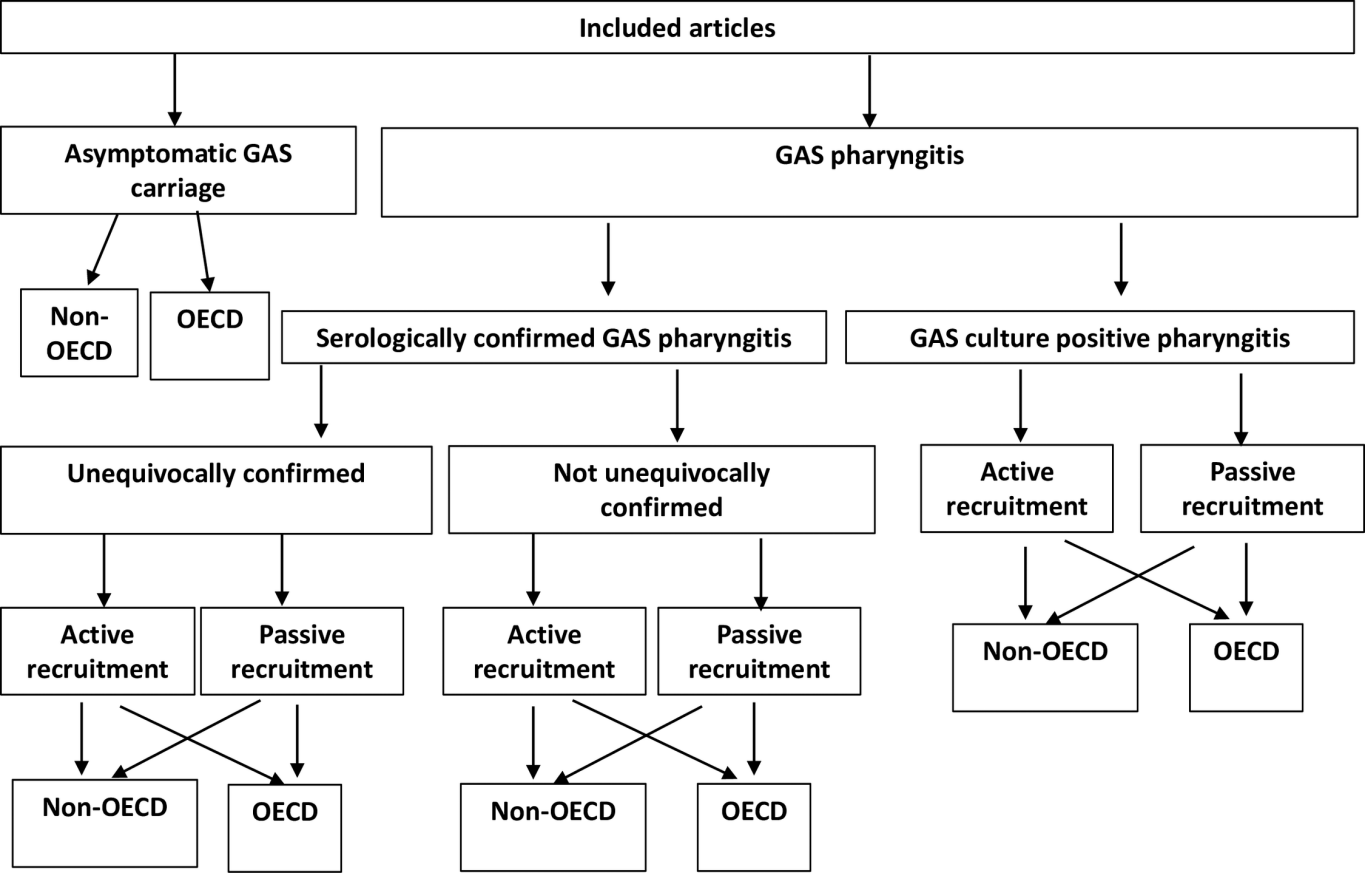
Article grouping system.

Results were stratified using up to five characteristics (Fig 1): (a) clinical outcome measured (GAS+ve pharyngitis, serologically-confirmed GAS pharyngitis, asymptomatic GAS carriage); (b) participant recruitment method (active or passive recruitment); (c) country income level (OECD or non-OECD member country); (d) age group; (e) where serologically-confirmed prevalence studies were reported, whether unequivocal case confirmation had occurred or otherwise.

‘GAS+ve pharyngitis’ was considered to occur when an individual with symptoms of pharyngitis or URTI received a throat swab which produced GAS when cultured. ‘Serologically-confirmed GAS pharyngitis’ was considered to occur when an individual with GAS+ve pharyngitis demonstrated an antibody reaction in response to GAS infection. ‘Unequivocal’ confirmation occurred when either a 0.2log_10_ or greater rise in ASO or ADB antibody titres was observed between acute and convalescent serum samples, or a four-fold increase in ASO titre occurred.[29, 33] ‘Asymptomatic GAS carriage’ occurred when individuals with no symptoms of pharyngitis or UTRI received a throat swab which produced GAS when cultured.

‘Passive recruitment’ was considered to occur where the study population presented to healthcare providers of their own accord and the practitioner obtained a throat swab. ‘Active recruitment’ occurred where a population had been sensitised to reporting pharyngitis or URTI symptoms to healthcare practitioners (e.g. by being asked about pharyngitis symptoms) and health services had been aligned to maximise accessibility to the participants (e.g. in terms of being close-by, involving home visits and/or offering tokens of thanks for study involvement). These distinctions were applied to pharyngitis studies. Prevalence studies of asymptomatic pharyngeal GAS carriage require active recruitment, as participants neither require nor present for treatment.

National Organisation for Economic Cooperation and Development (OECD) membership status was used as a means of classifying populations by socioeconomic position, as member countries tend to have high-income economies and very-high human development indexes[34] (Fig 1).

An ‘all ages’ analysis was performed, which included all studies in each category with no age restrictions applied. Other analyses were restricted to certain age groups: children aged <5 years old; children 5–19 years, all children aged < 20 years (‘children’) and ‘adults’ (generally including adults ≥20 years, but also allowing studies where this category started from ≥12 years if that was the adult category the authors used). If the study did not state the population age range, it was included in the ‘all ages’ analysis only. Exceptions were when the study population was described using terms such as ‘paediatric’, in which case it was pooled in the ‘Children’ category. The overall ‘children’ category included more studies than all the child subgroups put together. In order to be included in a more specific age category, the exact age range of the participants was required to be specified, and in many studies participants were simply described using terms such as ‘pediatric’ or ‘children under 16 years-old’. Similarly, studies which described their populations in such terms as ‘university students’ were grouped in the ‘Adult’ category.

### Statistical methods

Where individual articles did not state case data, but stated the prevalence of GAS and included the number of participants tested, the prevalence percentage was multiplied by the number of participants to find the number of cases.

A random-effects meta-analysis was used to produce pooled estimates for all outcome measures. Outcome measures were expressed as summary point prevalence percentages with 95% confidence intervals (CIs). Serologically confirmed GAS pharyngitis prevalence was calculated in two ways: serologically-confirmed patients as a proportion of the total number of symptomatic participants who had throat swabs; and as a proportion of those with GAS+ve throat swabs only. A sub-analysis of serological GAS studies was performed, including only those studies that met the unequivocal criteria. The programme R (version 3.4.1) was used throughout the analysis with the meta package.[35]

To estimate what proportion of total variation across study groups was due to heterogeneity rather than chance, the I^2^ statistic was used. Heterogeneity in pooled study groups was considered low if I^2^ <30%, moderate if 30–59%, substantial if 60–75% and considerable if >75%.[36]

## Results

### Search findings

**Fig 2:**
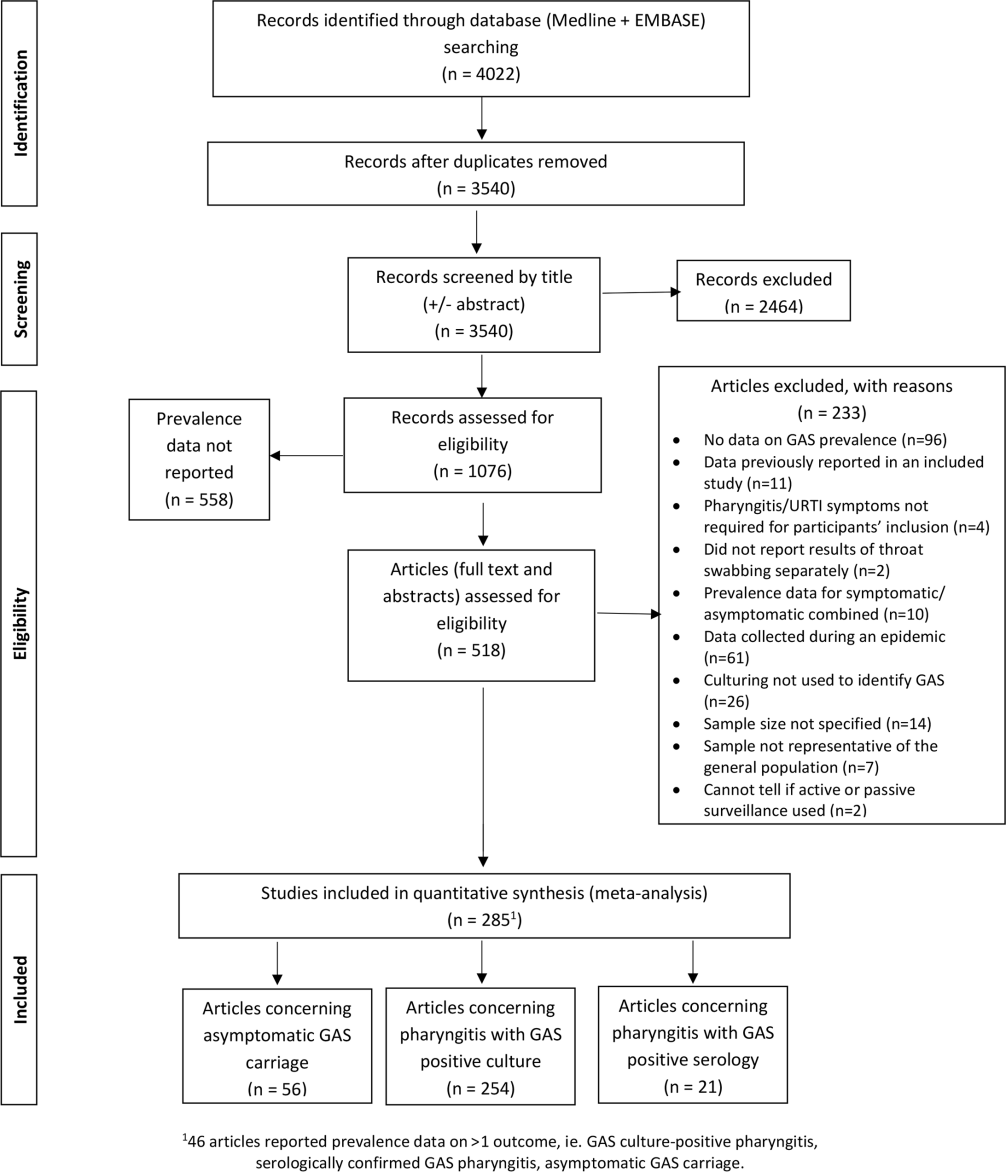
Results of literature search and study selection.

In total, 4,022 articles were identified and 1,076 were selected for further investigation. Exclusion criteria are listed in Fig 2. Overall, 285 articles that reported prevalence data on GAS were included.

### Included articles

Included articles addressed GAS culture-positive pharyngitis (254 studies), serologically-confirmed GAS pharyngitis (21 studies) and asymptomatic GAS carriage (56 studies). Of studies that reported on culture-positive pharyngitis, only 22% (57 studies) reported on populations that did not live in OECD countries. Nine of these 57 studies used active recruitment strategies, as did nine OECD studies. All others used passive recruitment. Three-quarters of passive recruitment studies were based in community primary care settings, often general practitioner clinics. One quarter were based in hospital Emergency Departments. Approximately 10% recruited in both hospital and primary care clinics.

Considerable heterogeneity was observed within most pooled study groups with some exceptions. Pooled serological studies demonstrated moderate heterogeneity, and low heterogeneity when pooled by age group. Moderate heterogeneity was also observed in the pooled prevalence estimate for GAS carriage in children <5 years old. Further details of abstracted data in this review are presented in the S1 Appendix, with accompanying measures of heterogeneity.

### GAS culture-positive pharyngitis

A detailed breakdown of prevalence estimates with numbers of included studies and participants is provided in Table 1.

**Table 1:** Prevalence of GAS culture-positive pharyngitis by age group, recruitment strategy and setting.

Population and Age group	Studies reporting prevalence data (No.)	GAS positive pharyngitis/ URTI patients (n)	Total pharyngitis/ URTI patients tested (N)	Prevalence of GAS positive patients (%)	95% CI
**OECD and non-OECD studies combined**					
***Active & Passive recruitment combined***					
<5 Years	24	1729	8960	16.6	12.6–21.6
5–19 Years	39	28348	222830	24.3	19.3–30.1
‘Children’	173	49143	315993	25.2	23.1–27.5
‘Adults’	57	15008	87834	13.7	11.1–16.8
All ages	254	83339	496288	22.7	21.2–24.2
**OECD studies**					
***Passive recruitment***					
<5 Years	17	859	5946	14.2	11.5–17.3
5–19 Years	18	3983	14279	36.8	30.9–43.1
‘Children’	120	20457	87164	28.5	26.3–30.8
‘Adults’	48	14794	86234	14.2	11.3–17.7
All ages	188	42740	199558	25.2	23.5–26.9
***Active recruitment***					
<5 Years	1	50	84	59.5	49.0–70.0
5–19 Years	5	20925	193231	11.6	8.3–16.1
‘Children’	6	21089	193707	16.6	11.9–22.7
‘Adults’	2	95	716	8.4	0.8–51.8
All ages	9	31831	254461	11.1	8.4–14.6
**Non-OECD studies**					
***Passive recruitment***					
<5 Years	6	820	2930	22.8	13.7–35.4
5–19 Years	7	2513	6679	37.4	27.7–48.2
‘Children’	38	6670	26481	23.1	19.7–26.8
‘Adults’	7	119	884	11.6	6.2–20.8
All ages	48	7841	33628	19.9	16.8–23.3
***Active recruitment***					
<5 Years	0	-	-	-	-
5–19 Years	9	927	8641	9.2	4.9–16.6
‘Children’	9	927	8641	9.2	4.9–16.6
‘Adults’	0	0	-	-	-
All ages	9	927	8641	9.2	4.9–16.6

NB: Although grouped within a specified age category (column 1), not all studies spanned the entire age range stated. For example, a study grouped in the 5-19-year-old analysis may have reported prevalence data for children aged 6–8 years old only.

For inclusion within a specified age category, the study must have explicitly reported prevalence data on people within this age group. Consequently, if the number of studies in the <5-year-old category are added to those in the 5-19-year-old category, the product may be less than the number of studies included the overall ‘Children’ category. This is because the ‘Children’ category also contains studies which recruited across both the <5-year-old and the 5-19-year-old age groups, as well as studies which only specified their participants’ age range broadly, using terms such as ‘pediatric’. The ‘all ages’ category includes all studies, regardless of whether the participants’ age range was described.

As no studies employed both passive and active recruitment, or were conducted in both OECD and non-OECD countries, totals in these columns will add up to the reported totals.

The overall ‘all age’ prevalence of GAS+ve pharyngitis was 22.7% (95% CI: 21.2–24.2%). Children (<20 years old) demonstrated the highest prevalence of culture-positive GAS pharyngitis: 25.2% (23.1–27.5%). When restricted to children aged <5 years old, a 16.6% (12.6–21.6%) prevalence was estimated. When restricted to children aged 5–19 years old, a prevalence of 24.3% (19.3–30.1%) was found. A prevalence of 13.7% (11.1–16.8%) was identified in adults. GAS+ve pharyngitis was more prevalent in OECD countries: 24.3% (22.6–26.1%) than in non-OECD countries: 17.6% (14.9–20.7%).

**Fig 3:**
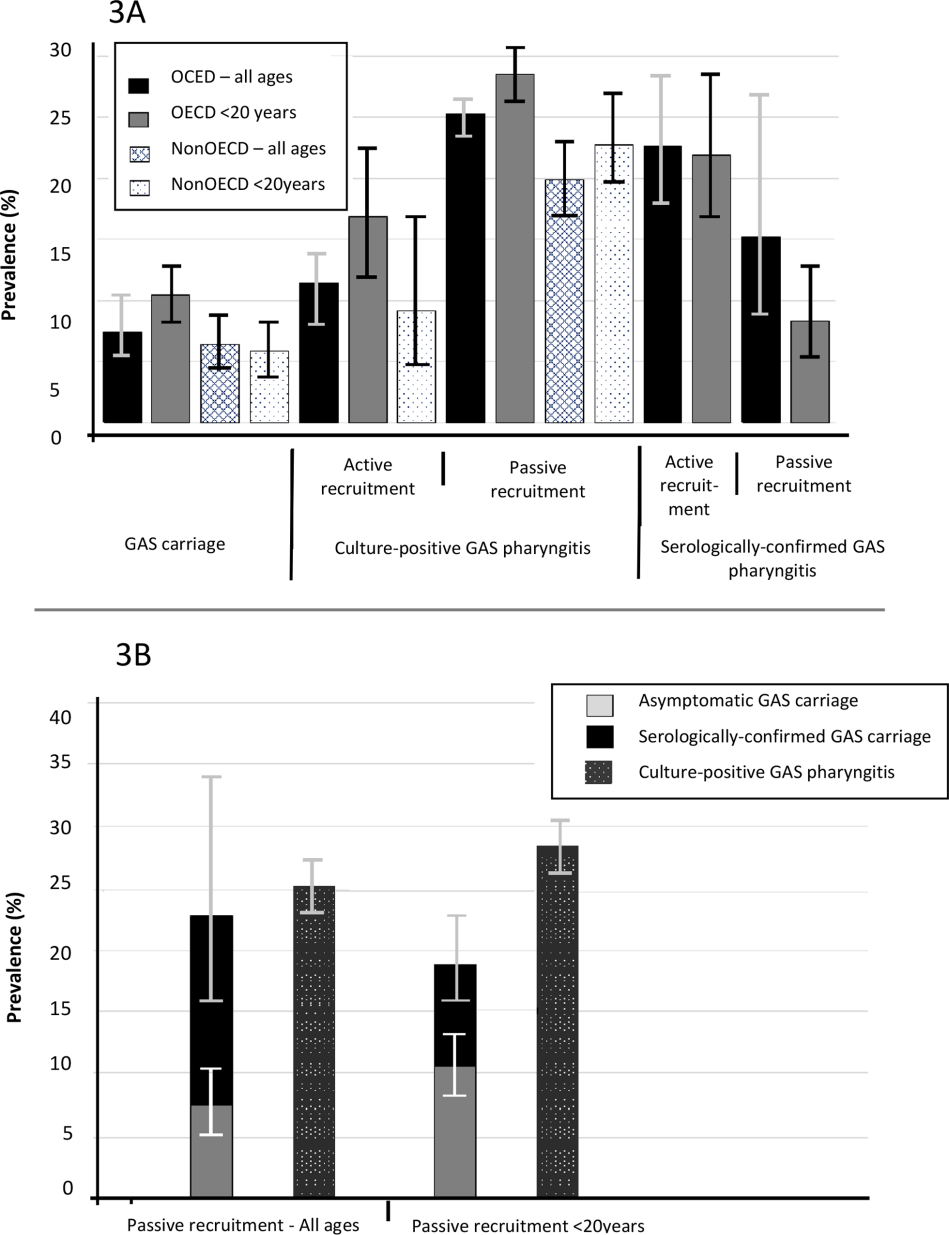
The relationship between different manifestations of pharyngeal GAS in OECD and non-OECD countries and active and passive recruitment settings (3A) and restricted to OECD countries in passive recruitment settings (3B).

Passive recruitment, that is clinical settings where participants self-present to a healthcare provider, generally detected markedly higher prevalence estimates (overall prevalence: 24.1%, 22.6–25.6%) than active recruitment (overall prevalence: 10.0%, 8.1–12.4%), where a population was sensitised to reporting a sore throat. This discrepancy was especially marked in 5-19-year-old OECD children (with a pooled prevalence of 36.8% (30.9–43.1%) in clinical settings, compared with 11.6% (8.3–16.1%) in active sore throat management programmes). Similarly, for non-OECD 5-19-year-old children, the prevalence of GAS+ve pharyngitis was much higher: 37.4% (27.7–48.2%) in clinical settings than in active sore throat management programmes: 9.2% (4.9–16.6%, Table 1, Fig 3).

### Serologically-confirmed GAS pharyngitis

This review attached most weight to the pooled prevalence estimate from six reported studies that used the unequivocal criteria for detecting serologically-confirmed GAS pharyngitis. Only 12 of 21 studies investigating serologically-confirmed GAS pharyngitis provided data on the total number of symptomatic individuals swabbed to identify those that were GAS+ve, on whom serological investigation was undertaken (details in S1 Appendix). Of these 12 studies, six reported using the unequivocal criteria–that being a significant titre increase in paired sera. All six were conducted in OECD populations and only one used active recruitment.

The overall ‘all age’ prevalence of serologically-confirmed GAS pharyngitis was 9.4% (5.6–15.5%). Studies using the unequivocal criteria detected a higher pooled prevalence (with an overall ‘all age’ prevalence of 16.4%, 9.9–26.0%). Higher prevalence estimates (22.6%, 17.8–28.2%) were detected when active recruitment was used, however this estimate is based on a single study which included paired serology. By comparison, pooled studies which used passive recruitment with unequivocal confirmation detected an overall prevalence of 15.2% (8.1–26.7%). Where participants have GAS+ve pharyngitis, the proportion of serologically-confirmed patients is around 50%, and around 60% in 5-19-year-old children (Table 2, Fig 3).

**Table 2:** Total unequivocal serologically-confirmed GAS pharyngitis prevalence by age group, recruitment strategy and setting, including where GAS culture positive swabs were obtained.

Population age group	Studies reporting prevalence data included (No.)	Serologically confirmed GAS pharyngitis cases (n)	Total pharyngitis/ URTI cases tested (N)	Prevalence of confirmed GAS pharyngitis (%)	95% CI	Culture positive GAS pharyngitis cases (n)	Prevalence of culture positive GAS pharyngitis (%)	95% CI	Prevalence of serologically confirmed GAS pharyngitis in GAS positive cultures (%)	95% CI
**OECD studies**										
***Active & Passive recruitment***										
<5 Years	3	47	504	10.0	5.5–17.5	88	17.9	12.7–24.6	53.3	42.3–63.9
5–19 Years	2	44	304	12.34	3.06–38.58	72	23.7	19.3–28.8	61.1	49.4–71.6
‘Children’	5	258	2168	10.3	6.6–15.7	450	18.8	13.1–26.1	57.1	51.9–62.2
‘Adults’	1	14	57	24.6	15.2–37.1	20	35.1	22.6–47.4	70.0	48.1–85.5
All ages	6	390	2490	16.4	9.9–26.0	720	34.1	19.1–53.2	53.3	47.0–59.6
***Passive recruitment***										
<5 Years	2	40	467	7.9	4.3–13.8	78	16.1	11.6–22.0	51.3	40.3–62.2
5–19 Years	1	9	150	6.0	2.2–9.8	16	10.7	5.7–15.6	56.3	31.9–80.6
‘Children’	4	216	1976	8.4	5.4–12.8	384	15.9	11.1–22.1	56.0	50.5–61.3
‘Adults’	0	-	-	-	-	-	-	-	-	-
All ages	5	334	2242	15.2	8.1–26.7	634	34.0	16.4–57.6	51.3	45.4–57.3
***Active recruitment***										
<5 Years	1	7	37	18.9	9.5–34.2	10	27.0	12.7–41.3	70.0	39.7–89.2
5–19 Years	1	35	154	22.7	16.8–30.0	56	36.4	28.8–44.0	62.5	49.4–74.0
‘Children’	1	42	192	21.9	16.6–28.3	66	34.6	27.8–41.3	63.6	51.6–74.2
‘Adults’	1	14	57	24.6	15.2–37.1	20	35.1	22.6–47.4	70.0	48.1–85.5
All ages	1	56	248	22.6	17.8–28.2	86	34.7	29.0–40.8	65.1	54.6–74.4

NB: Although grouped within a specified age category (column 1), not all studies spanned the entire age range stated. For example, a study grouped in the 5-19-year-old analysis may have reported prevalence data for children aged 6–8 years old only.

For inclusion within a specified age category, the study must have explicitly reported prevalence data on people within this age group. Consequently if the number of studies in the <5-year-old category are added to those in the 5-19-year-old category, the product may be less than the number of studies included the overall ‘Children’ category. This is because the ‘Children’ category also contains studies which recruited across both the <5-year-old and the 5-19-year-old age groups, as well as studies which only specified their participants’ age range broadly, using terms such as ‘pediatric’. The ‘all ages’ category includes all studies, regardless of whether the participants’ age range was described.

As no studies employed both passive and active recruitment, totals in these columns will add up to the reported totals.

### Asymptomatic GAS carriage

Table 3 shows the prevalence of GAS carriage as well as numbers of included studies and participants. The overall prevalence of asymptomatic carriage was 7.0% (5.6–8.8%). When studies were pooled regardless of country income, the highest carriage was observed in children <20 years old (8.0%, 6.6–9.7%). A slightly lower overall prevalence was detected in non-OECD settings (6.4%, 4.6–8.9%, compared with 7.5%, 5.3–10.3%) in OECD settings (Table 3, Fig 3).

**Table 3:** The prevalence of GAS carriage by age group, recruitment strategy and setting.

Population Age group	Studies reporting prevalence data included (No.)	Pharyngeal GAS carriers (n)	Total people throat swabbed (N)	Prevalence of GAS positive cases (%)	95% CI
**OCED & non-OECD studies combined**					
<5 Years	7	27	1286	2.8	1.5–5.3
5–19 Years	23	1529	19997	7.9	0.6–10.9
‘Children’	46	3211	39486	8.0	6.6–9.7
‘Adults’	12	367	14756	2.8	1.5–5.0
All ages	56	4055	59801	7.0	5.6–8.8
**OECD studies**					
<5 Years	6	27	1160	3.1	1.6–5.8
5–19 Years	11	454	4211	11.2	8.2–15.2
‘Children’	26	1148	11051	10.5	8.4–12.9
‘Adults’	9	274	12726	2.0	0.8–5.0
All ages	34	1658	27982	7.5	5.3–10.3
**Non-OECD studies**					
<5 Years	1	0	126	0.4	0.0–0.6
5–19 Years	12	1075	15786	5.6	3.2–9.5
‘Children’	20	2063	28435	5.9	4.3–8.1
‘Adults’	3	93	2030	4.6	3.8–5.6
All ages	22	2397	31819	6.4	4.6–8.9

NB: Although grouped within a specified age category (column 1), not all studies spanned the entire age range stated. For example, a study grouped in the 5-19-year-old analysis may have reported prevalence data for children aged 6–8 years old only.

For inclusion within a specified age category, the study must have explicitly reported prevalence data on people within this age group. Consequently if the number of studies in the <5-year-old category are added to those in the 5-19-year-old category, the product may be less than the number of studies included the overall ‘Children’ category. This is because the ‘Children’ category also contains studies which recruited across both the <5-year-old and the 5-19-year-old age groups, as well as studies which only specified their participants’ age range broadly, using terms such as ‘pediatric’. The ‘all ages’ category includes all studies, regardless of whether the participants’ age range was described.

As no studies employed both passive and active recruitment, totals in these columns will add up to the reported totals, however.

### Comparison of GAS outcomes across populations and settings

Pooled prevalence of GAS+ve pharyngitis, serologically confirmed pharyngitis and asymptomatic carriage are shown graphically in Fig 3A–3B for specific age groups and country income levels.

GAS+ve pharyngitis was the most prevalent manifestation of GAS. Higher levels were found in OECD countries. The overall prevalence of carriage was similar in high- and low-country income settings, however GAS carriage was twice as prevalent in children from OECD countries compared to children in non-OECD countries (Fig 3A). In passive recruitment OECD studies overall, the sum of the asymptomatic carriage prevalence and the serologically confirmed GAS pharyngitis prevalence approximately equals the prevalence of culture-positive GAS pharyngitis. This relationship was also observed, albeit with less certainty, when restricted to children <20 years old (Fig 3B). This relationship could not be explored in active recruitment settings as only one study in that category examined serologically confirmed GAS pharyngitis.

## Discussion

To our knowledge, this is the first comprehensive review of pharyngeal GAS detection that has assessed all three of its clinically relevant manifestations: GAS+ve pharyngitis; serologically-confirmed GAS pharyngitis; and asymptomatic GAS carriage. The prevalence of serologically-confirmed GAS pharyngitis for school-aged children, who have the highest risk of ARF, has been quantified. In high-income countries only one in 10 children with pharyngitis symptoms are likely to have serologically-confirmed GAS pharyngitis. Where participants are identified as having GAS +ve pharyngitis, the proportion that are serologically-confirmed is around 50–60%. This finding supports the use of throat swabbing in symptomatic children, rather than providing presumptive antibiotic treatment. The prevalence of serologically-confirmed GAS pharyngitis indicates how many children may go on to develop ARF as a complication,[19, 23, 37] which in turn indicates how effective a sore throat management programme is likely to be. A limitation here, as with all sore-throat management programmes, is that up to two-thirds of ARF cases do not appear to present with preceding pharyngitis, so other prevention strategies are necessary when seeking to remove the burden of disease.[38, 39]

The decision to obtain a throat swab was likely influenced by the healthcare practitioner’s suspicion for GAS pharyngitis and concern for possible complications. The Centor criteria gives an indication of the likelihood of a sore throat being due to bacterial infection. Practitioners may have been more inclined to swab patients presenting with symptoms suggestive of GAS pharyngitis, such as fever.[40, 41] GAS+ve prevalence is strongly affected by whether patients present to sore throat management programmes that actively recruit them (active recruitment), or present of their own accord to healthcare practitioners with manifestations of pharyngitis (passive recruitment). Passive recruitment strategies tend to detect a higher prevalence, compared with active recruitment methods–for example, in children less than 20 years old, a higher prevalence (28.5% in OECD countries and 23.1% in non-OECD countries) was observed in those presenting to primary healthcare providers, compared with those identified through specialised programmes (16.6% in OECD countries and 9.1% in non-OECD countries). This difference may be due to active recruitment studies including patients with less severe pharyngitis, who would not otherwise seek treatment for their symptoms. Given that an estimated 8% of children are carriers, if the prevalence of GAS detection in active recruitment studies approaches this level, then the majority of culture-positive patients are likely to have carriage, not true GAS pharyngitis. It is therefore important for active sore throat treatment programmes to monitor the prevalence of GAS detection and consider serological testing for a sample of patients.

Around 37% of 5-19-year-old children in passive recruitment settings have GAS+ve pharyngitis, both in OECD and non-OECD countries. Despite this, many non-OECD countries have much higher rates of GAS diseases and ARF.[1] The intercountry distribution of GAS pharyngitis does not therefore appear to reflect the very wide difference in ARF rates. This apparent discrepancy could be because GAS skin infections dominate in tropical climates where the highest burden of contemporary ARF occurs and may be a major driver of ARF in these settings.[42–44] ARF is also ecologically associated with poverty, overcrowding and potentially other environmental factors which vary markedly in time and place.[45, 46] International research has consistently noted associations between ARF and socioeconomic conditions, including poor housing conditions.[1, 45, 47–54]

Our review of 285 studies greatly extends the findings of a previous review by Shaikh *et al*. which included only 29 studies[24] and did not include data on GAS seroconversion, which is generally accepted as the key clinical outcome that triggers the autoimmune process driving ARF.[23, 37, 55] Shaikh *et al*. also reported a pooled prevalence estimate of 37% for GAS culture-positive pharyngitis in children, the same as our identified prevalence for 5–19 year-old children in passive recruitment settings (where the majority of included studies originated). The use of a less stringent inclusion criteria to meet our study aims is justified as sore throat management programmes will generally treat any GAS+ve patient with a self-reported sore throat.[56] The previous review reported a 12% asymptomatic carriage prevalence estimate, very similar to our carriage estimate for OECD children, but higher than our overall carriage estimate for children of 8%.

This review has several limitations. Firstly, as pooled studies span multiple continents, ecological bias is apparent. Pooling study data over time averages temporal variation in GAS distribution. Thus it is not possible to distinguish geographical or temporal trends in the pooled prevalence estimates. Stratifying according to OECD status was intended to reduce these effects, particularly that of disease determinants and ecological biases. Secondly, whether ASO and ADB titres are a valid means of identifying ‘true’ GAS pharyngitis remains a matter of debate, however an increase in antibody titre is a much more accurate indicator of GAS infection than a single titre result.[57] Serological techniques to determine titre may have varied across studies. Our use of the unequivocal criteria for serological confirmation attempted to minimise these biases. Thirdly, despite our best efforts, it is possible that relevant literature was not identified or was mistakenly excluded as our database access permitted articles dating back to 1946 to be obtained online. Finally, we did not attempt to systematically assess publication bias or the quality of included studies (beyond investigating heterogeneity). Hand-searches of previously published review reference lists were performed in an attempt to reduce publication bias. Risk of bias assessment tools can be problematic due to the broad nature of their specifications and the variable interpretation (and applicability) of criteria.[58] Studies in closed communities and disease outbreak settings were excluded in an attempt to reduce selection bias, as was our use of stringent inclusion criteria, and stratifying findings according to participants’ age range and country income level. Despite this, there is still considerable heterogeneity in most pooled study categories. This is likely due to differences in inclusion and exclusion criteria applied within the pooled studies. As a result, a range of clinical manifestations and severities are included in the final prevalence calculations. This feature can actually be considered a study strength, given the wide range of clinical presentations pharyngitis patients present to healthcare practitioners with.

### Summary

Due to the collateral damage of antibiotic misuse on human health and the environment, there is a pressing need to target pharyngitis testing and treatment in the most effective and efficient way possible. School-aged children with symptomatic sore throats have a relatively low chance of having serologically-confirmed GAS pharyngitis, particularly in organised sore throat management programmes. ARF prevention programmes need to be carefully designed with this knowledge in mind and targeted to groups at high risk of ARF. Ultimately, reducing ARF is likely to depend on prevention programmes that address the underlying determinants of disease risk, such as income, housing conditions and access to primary healthcare. Further research should validate the main conclusions of this systematic review, particularly through collection of GAS serological data in low- and middle-income countries. It would also be useful to have more studies that measured all three clinically important GAS throat infection outcomes in the same populations at the same time to see how these states are related to one another.

## Supporting information

S1 Checklist PRISMA checklist. (DOC)

S1 Appendix List of included studies, abstracted data and heterogeneity summaries. (XLSX)

## References

[1] Carapetis JR, Steer AC, Mulholland EK, Weber M. The global burden of group A streptococcal diseases. The Lancet Infectious Diseases. 2005;5(11):685–94. doi: 10.1016/S1473-3099(05)70267-X 16253886 10.1016/S1473-3099(05)70267-X

[2] Marx J. Rosen's emergency medicine: concepts and clinical practice. 7th ed Philadelphia, Pennsylvania:: Mosby/Elsevier; 2010.

[3] Little P, Stuart B, Hobbs FD, Butler CC, Hay AD, Campbell J, et al Predictors of suppurative complications for acute sore throat in primary care: prospective clinical cohort study. Bmj. 2013;347:f6867 doi: 10.1136/bmj.f6867 ; PubMed Central PMCID: PMCPMC3898431. 24277339 10.1136/bmj.f6867 PMC3898431

[4] Matthys J. There are still problems in identifying who will develop complications of sore throat in primary care. Bmj. 2014;348:g299 doi: 10.1136/bmj.g299 . 24448231 10.1136/bmj.g299

[5] Zwart S, Rovers MM, de Melker RA, Hoes AW. Penicillin for acute sore throat in children: randomised, double blind trial. BMJ. 2003;327(7427):1324 doi: 10.1136/bmj.327.7427.1324 ; PubMed Central PMCID: PMCPMC286321. 14656841 10.1136/bmj.327.7427.1324 PMC286321

[6] Ralph AP, Carapetis JR. Group A streptococcal diseases and their global burden Host-Pathogen Interactions in Streptococcal Diseases: Springer; 2013 p. 1–27. 10.1007/82_2012_280 23242849

[7] Carapetis JR, McDonald M, Wilson NJ. Acute rheumatic fever. Lancet. 2005;366(9480):155–68. doi: 10.1016/S0140-6736(05)66874-2 . 16005340 10.1016/S0140-6736(05)66874-2

[8] Marijon E, Mirabel M, Celermajer DS, Jouven X. Rheumatic heart disease. Lancet. 2012;379(9819):953–64. PubMed PMID: WOS:000301353700036. doi: 10.1016/S0140-6736(11)61171-9 22405798 10.1016/S0140-6736(11)61171-9

[9] Oliver J, Pierse N, Baker MG, Carapetis J. Comparison of approaches to rheumatic fever surveillance across Organisation for Economic Co-operation and Development countries. Journal of Pediatrics and Child Health. 2015;51(11): 1071–1077. 10.1111/jpc.12969 26174709

[10] Webb RH, Grant C, Harnden A. Acute rheumatic fever. Bmj. 2015;351:h3443 doi: 10.1136/bmj.h3443 . 26175053 10.1136/bmj.h3443

[11] McCarthy M. Most sore throats in US are still treated with antibiotics. Bmj. 2013;347:f6056 doi: 10.1136/bmj.f6056 . 24124182 10.1136/bmj.f6056

[12] Butler CC, Rollnick S, Pill R, Maggs-Rapport F, Stott N. Understanding the culture of prescribing: qualitative study of general practitioners' and patients' perceptions of antibiotics for sore throats. Bmj. 1998;317(7159):637–42. ; PubMed Central PMCID: PMCPMC28658. 9727992 10.1136/bmj.317.7159.637 PMC28658

[13] Kumar S, Little P, Britten N. Why do general practitioners prescribe antibiotics for sore throat? Grounded theory interview study. Bmj. 2003;326(7381):138 ; PubMed Central PMCID: PMCPMC140007. 12531847 10.1136/bmj.326.7381.138 PMC140007

[14] Fleetcroft R. Length of penicillin treatment of streptococcal infections. Antibiotics should not be used for self limiting illnesses. BMJ. 2000;320(7250):1665; author reply 6–7. . 10905828

[15] Chiappini E, Regoli M, Bonsignori F, Sollai S, Parretti A, Galli L, et al Analysis of different recommendations from international guidelines for the management of acute pharyngitis in adults and children. Clin Ther. 2011;33(1):48–58. doi: 10.1016/j.clinthera.2011.02.001 . 21397773 10.1016/j.clinthera.2011.02.001

[16] Danchin MH, Curtis N, Nolan TM, Carapetis JR. Treatment of sore throat in light of the Cochrane verdict: Is the jury still out? Med J Aust. 2002;177(9):512–5. . 12405896 10.5694/j.1326-5377.2002.tb04925.x

[17] Kerdemelidis M, Lennon D, Arroll B, Peat B. Guidelines for sore throat management in New Zealand. ‎N Z Med J. 2009;122(1301):10–8. . 19829387

[18] Matthys J, De Meyere M, van Driel ML, De Sutter A. Differences among international pharyngitis guidelines: not just academic. The Annals of Family Medicine. 2007;5(5):436–43. doi: 10.1370/afm.741 17893386 10.1370/afm.741 PMC2000301

[19] Heart Foundation of New Zealand. Group A Streptococcal Sore Throat Management Guideline. 2014 Update. Auckland, Heart Foundation of New Zealand 2014.

[20] De Meyere M, Bogaert M, Verschraegen G, Mervielde I. Trial of prescribing strategies in managing sore throat. Penicillin had no effect in patients negative for group A beta haemolytic streptococci. Bmj. 1997;314(7098):1904–5. ; PubMed Central PMCID: PMCPMC2127000. 9224149 10.1136/bmj.314.7098.1904a PMC2127000

[21] Antibiotics for acute group A streptococcal pharyngitis. Prescrire Int. 2004;13(74):227–32. . 15612147

[22] Gerber MA, Randolph MF, Mayo DR. The group A streptococcal carrier state. A reexamination. Am J Dis Child. 1988;142(5):562–5. . 3128949 10.1001/archpedi.1988.02150050100043

[23] Shulman ST, Bisno AL, Clegg HW, Gerber MA, Kaplan EL, Lee G, et al Clinical practice guideline for the diagnosis and management of group A streptococcal pharyngitis: 2012 update by the Infectious Diseases Society of America. Clin Infect Dis. 2012;55(10):1279–82. doi: 10.1093/cid/cis847 . 23091044 10.1093/cid/cis847

[24] Shaikh N, Leonard E, Martin JM. Prevalence of streptococcal pharyngitis and streptococcal carriage in children: a meta-analysis. Pediatrics. 2010;126(3):e557–64. doi: 10.1542/peds.2009-2648 . 20696723 10.1542/peds.2009-2648

[25] Esposito S, Blasi F, Bosis S, Droghetti R, Faelli N, Lastrico A, et al Aetiology of acute pharyngitis: the role of atypical bacteria. J Clin Microbiol. 2004;53(Pt 7):645–51. doi: 10.1099/jmm.0.05487-0 . 15184536 10.1099/jmm.0.05487-0

[26] Danchin MH, Rogers S, Kelpie L, Selvaraj G, Curtis N, Carlin JB, et al Burden of acute sore throat and group A streptococcal pharyngitis in school-aged children and their families in Australia. Pediatrics. 2007;120(5):950–7. doi: 10.1542/peds.2006-3368 . 17974731 10.1542/peds.2006-3368

[27] Merlini AB, Stocco CS, Schafranski MD, Arruda P, Bail L, Borges CL, et al Prevalence of group A beta-hemolytic Streptococcus oropharyngeal colonization in children and therapeutic regimen based on antistreptolysin levels: Data from a city from Southern Brazil. Open Rheumatol J. 2014;8(1):13–7. doi: 10.2174/1874312901408010013 . 25136388 10.2174/1874312901408010013 PMC4136371

[28] DeMuri GP, Wald ER. The Group A Streptococcal Carrier State Reviewed: Still an Enigma. J Pediatric Infect Dis Soc. 2014;3(4):336–42. doi: 10.1093/jpids/piu030 . 26625454 10.1093/jpids/piu030

[29] Parks T, Smeesters PR, Curtis N, Steer AC. ASO titer or not? When to use streptococcal serology: a guide for clinicians. Eur J Clin Microbiol Infect Dis. 2015;34(5):845–9. doi: 10.1007/s10096-014-2303-8 PubMed PMID: WOS:000353470600001. 25560708 10.1007/s10096-014-2303-8

[30] Moher D, Liberati A, Tetzlaff J, Altman DG. Preferred reporting items for systematic reviews and meta-analyses: the PRISMA statement. Ann Intern Med. 2009;151(4):264–9. 19622511 10.7326/0003-4819-151-4-200908180-00135

[31] Shaikh N, Leonard E, Martin JM. Prevalence of streptococcal pharyngitis and streptococcal carriage in children: a meta-analysis. Pediatrics. 2010;126(3):e557–e64. doi: 10.1542/peds.2009-2648 20696723 10.1542/peds.2009-2648

[32] Choby BA. Diagnosis and treatment of streptococcal pharyngitis. Am Fam Physician. 2009;79(5):383–90. . 19275067

[33] Steer AC, Smeesters PR, Curtis N. Streptococcal Serology: Secrets for the Specialist. Pediatr Infect Dis J. 2015;34(11):1250–2. doi: 10.1097/INF.0000000000000881 26270790 10.1097/INF.0000000000000881

[34] OECD.org. Oragnisation for Economic Development and Cooperation (OECD) Paris2016 [cited 2016 6 May]. Available from: www.oecd.org/.

[35] R core team. R: A language and environment for statistical computing. R Foundation for Statistical Computing, Vienna, Austria http://www.R-project.org/. 2013.

[36] Klem F, Wadhwa A, Prokop LJ, Sundt WJ, Farrugia G, Camilleri M, et al Prevalence, Risk Factors, and Outcomes of Irritable Bowel Syndrome After Infectious Enteritis: A Systematic Review and Meta-analysis. Gastroenterology. 2017;152(5):1042–54 e1. doi: 10.1053/j.gastro.2016.12.039 ; PubMed Central PMCID: PMCPMC5367939. 28069350 10.1053/j.gastro.2016.12.039 PMC5367939

[37] Kaplan EL, Gastanaduy AS, Huwe BB. The role of the carrier in treatment failures after antibiotic therapy for group A streptococci in the upper respiratory tract. J Lab Clin Med. 1981;98(3):326–35. . 7021717

[38] Veasy LG, Wiedmeier SE, Orsmond GS, Ruttenberg HD, Boucek MM, Roth SJ, et al Resurgence of acute rheumatic fever in the intermountain area of the United States. N Engl J Med. 1987;316(8):421–7. doi: 10.1056/NEJM198702193160801 . 3807984 10.1056/NEJM198702193160801

[39] Noonan S, Zurynski YA, Currie BJ, McDonald M, Wheaton G, Nissen M, et al A national prospective surveillance study of acute rheumatic fever in Australian children. Pediatr Infect Dis J. 2013;32(1):e26–32. doi: 10.1097/INF.0b013e31826faeb3 . 22926211 10.1097/INF.0b013e31826faeb3

[40] Centor RM, Witherspoon JM, Dalton HP, Brody CE, Link K. The diagnosis of strep throat in adults in the emergency room. Med Decis Making. 1981;1(3):239–46. doi: 10.1177/0272989X8100100304 . 6763125 10.1177/0272989X8100100304

[41] Wald ER, Green MD, Schwartz B, Barbadora K. A streptococcal score card revisited. Pediatr Emerg Care. 1998;14(2):109–11. . 9583390 10.1097/00006565-199804000-00005

[42] McDonald M, Currie BJ, Carapetis JR. Acute rheumatic fever: a chink in the chain that links the heart to the throat? The Lancet Infectious Diseases. 2004;4(4):240–5. doi: 10.1016/S1473-3099(04)00975-2 . 15050943 10.1016/S1473-3099(04)00975-2

[43] Currie BJ, Carapetis JR. Skin infections and infestations in Aboriginal communities in northern Australia. Australas J Dermatol. 2000;41(3):139–43; quiz 44–5. . 10954983 10.1046/j.1440-0960.2000.00417.x

[44] Parks T, Smeesters PR, Steer AC. Streptococcal skin infection and rheumatic heart disease. Curr Opin Infect Dis. 2012;25(2):145–53. doi: 10.1097/QCO.0b013e3283511d27 . 22327467 10.1097/QCO.0b013e3283511d27

[45] Jaine R, Baker M, Venugopal K. Acute rheumatic fever associated with household crowding in a developed country. Pediatr Infect Dis J. 2011;30(4):315–9. doi: 10.1097/INF.0b013e3181fbd85b . 20948456 10.1097/INF.0b013e3181fbd85b

[46] Steer AC. Historical aspects of rheumatic fever. J Paediatr Child Health. 2015;51(1):21–7. doi: 10.1111/jpc.12808 . 25586841 10.1111/jpc.12808

[47] White H, Walsh W, Brown A, Riddell T, Tonkin A, Jeremy R, et al Rheumatic heart disease in indigenous populations. Heart Lung Circ. 2010;19(5–6):273–81. doi: 10.1016/j.hlc.2010.02.019 . 20356783 10.1016/j.hlc.2010.02.019

[48] Carapetis JR, Carapetis JR. Rheumatic heart disease in Asia. Circulation. 2008;118(25):2748–53. doi: 10.1161/CIRCULATIONAHA.108.774307 . 19106399 10.1161/CIRCULATIONAHA.108.774307

[49] Omurzakova NA, Yamano Y, Saatova GM, Mirzakhanova MI, Shukurova SM, Kydyralieva RB, et al High incidence of rheumatic fever and rheumatic heart disease in the republics of Central Asia. Int J Rheum Dis. 2009;12(2):79–83. doi: 10.1111/j.1756-185X.2009.01388.x . 20374323 10.1111/j.1756-185X.2009.01388.x

[50] Gordis L. The virtual disappearance of rheumatic fever in the United States: lessons in the rise and fall of disease. T. Duckett Jones memorial lecture. Circulation. 1985;72(6):1155–62. 4064266 10.1161/01.cir.72.6.1155

[51] Vendsborg P, Hansen LF, Olesen KH. Decreasing incidence of a history of acute rheumatic fever in chronic rheumatic heart disease. Cardiologia. 1968;53(6):332–40. . 5745504 10.1159/000166204

[52] Oliver J, Pierse N, Jackson C, Baker MG. The housing conditions of rheumatic fever cases in New Zealand: A descriptive study A report commissioned by the New Zealand Ministry of Health. Wellington: University of Otago, Wellington, 2015.

[53] Zaman MM, Yoshiike N, Chowdhury AH, Jalil MQ, Mahmud RS, Faruque GM, et al Socio-economic deprivation associated with acute rheumatic fever. A hospital-based case-control study in Bangladesh. Paediatr Perinat Epidemiol. 1997;11(3):322–32. . 9246693 10.1111/j.1365-3016.1997.tb00011.x

[54] Vlajinac H, Adanja B, Marinkovic J, Jarebinski M. Influence of socio-economic and other factors on rheumatic fever occurrence. Eur J Epidemiol. 1991;7(6):702–4. . 1783067 10.1007/BF00218687

[55] Heart Foundation of New Zealand. New Zealand Guidelines for Rheumatic Fever: Diagnosis, Management and Secondary Prevention of Acute Rheumatic Fever and Rheumatic Heart Disease: 2014 Update. 2014.

[56] World Health Organization. WHO Model Prescribing Information: Drugs Used in the Treatment of Streptococcal Pharyngitis and Prevention of Rheumatic Fever. Geneva: 1999.

[57] Johnson DR, Kurlan R, Leckman J, Kaplan EL. The human immune response to streptococcal extracellular antigens: clinical, diagnostic, and potential pathogenetic implications. Clin Infect Dis. 2010;50(4):481–90. doi: 10.1086/650167 20067422 10.1086/650167

[58] Katrak P, Bialocerkowski AE, Massy-Westropp N, Kumar S, Grimmer KA. A systematic review of the content of critical appraisal tools. BMC Med Res Methodol. 2004;4:22 doi: 10.1186/1471-2288-4-22 ; PubMed Central PMCID: PMCPMC521688. 15369598 10.1186/1471-2288-4-22 PMC521688

